# Complex thinking and social entrepreneurship. An approach from the methodology of compositional data analysis

**DOI:** 10.1016/j.heliyon.2023.e13415

**Published:** 2023-02-03

**Authors:** Marco Cruz-Sandoval, José Carlos Vázquez-Parra, Martina Carlos-Arroyo

**Affiliations:** Institute for the Future of Education, Tecnologico de Monterrey, Monterrey, Mexico

**Keywords:** Professional education, Educational innovation, Future of education, Complex thinking, Compositional data analysis, Higher education, Log-ratio, CoDa

## Abstract

This article describes a study that sought to identify the correlation between social entrepreneurship and complex thinking competencies in a population of Mexican students. The article uses the novel approach of compositional data analysis. Compositional data analysis focuses on studying data that are part of a whole, whose importance lies in the relative information between its parts and not in absolute values. The analysis allows us to identify an association between the competencies and find statistically significant relationships between sub-competencies. In particular, correlations can be seen between the sub-competencies of scientific thinking and critical thinking with the sub-competency of self-control, leadership, and social awareness and social value. Furthermore, a correlation is observed between systemic thinking with the sub-competency of social awareness and social value, self-control, and leadership sub-competencies. In a practical way, this article introduces the compositional data analysis methodology to the academic educational literature focused on the study and measurement of competencies, opening possibilities for more precise, broad, descriptive analyses not achieved with other methodologies.

## 1 Introduction

One of the most significant challenges facing any contemporary university is not acquiring knowledge or developing competencies but instead designing educational models that combine this knowledge or competencies to resolve practical problems of professional life [1]. In other words, it is not enough to acquire tools; efforts must also be made to ensure that future professionals know how to use them integratively in real situations [2]. Thus, it is increasingly common to find correlational studies that analyze how competencies synchronize, intending to find alternative ways to optimize the training of new professionals [3].

Something noteworthy is that studies that measure the development and achievement of competencies in education are usually focused on identifying the statistical relationship between two variables that are commonly expressed as a percentage [[4], [5], [6], [7]]. In these studies, the data represent parts of a whole, which subjects the results to the restriction that the sum of the parts must be unity (the whole) or a constant. This could lead to spurious relationships and erroneous conclusions in the studies [[8], [9], [10]].

Therefore, this article proposes adopting a compositional data analysis methodology when performing correlation studies of formative competencies, considering that they allow appreciating the importance of the relative information of the parts and not absolute values. In this sense, the analysis aims to show the relative importance between the different sub-competencies that constitute two different educational formative competencies. As an example of analysis, we offer data collected from a study that aims to show the statistical correlation in the perception of achievement that students have in the development of the sub-competencies of: Social Entrepreneurship and Complex Thinking competencies. The results derive from an educational intervention applied to a group of first-semester university students in a class where a social entrepreneurship project was devised.

## 2 Theoretical framework

### 2.1 About the formation of competencies

Competency training is related to identifying those characteristics that an individual requires to carry out a task, considering the knowledge, skills, and attitudes involved to fulfill its objective efficiently and optimally [11]. According to Tobón [12], the acquisition and development of competencies should involve a horizon of integrated skills combining theory and practice in the work to be undertaken. Thus, competencies must be valued as combinatory knowledge, in which the learners acquire priority significance since they convert knowledge into know-how [13].

Currently, in a globalized, fluid, and uncertain world, training to develop competencies becomes a primary issue for universities because, while the knowledge acquired may lag the exponential evolution of research and science, the skills developed in the implementation process advance with the development of the individual, both in their educational process and professional training [14]. The comprehensive nature of competencies allows future professionals to develop skills that mobilize various elements to deal with a challenge, decision, or problem, making them more flexible in addressing the needs of a changing world [15].

However, it is essential to mention that measuring the achievement or performance of competencies is not a simple task to be approached lightly since the characteristic of integrality in a competency requires paying attention to its general development and also the balanced performance of its sub-competencies, indicators, or domains, and the individual's perception, which can be influenced by their social, economic, and cultural characteristics [16]. If the methodology used is too rigid or strict with the generalization of the data, the information could be lost and, with it, a deeper insight into the very process of acquisition and development of the competency by failing to consider particular characteristics of the sample [17].

The relevance of using an analysis methodology that avoids spurious results through the analysis of relative information such as compositional data analysis, enables the measurement of achievement profile, progress, or perception of competent performance in such a way that educational institutions can build a formative curriculum plan that strengthens the development of competencies and sub-competencies addressing the needs of a complex world in which the achievement of their students is considered to be deficient.

### 2.2 Social entrepreneurship and complex thinking competencies

Conceptually, the competence of complex thinking involves the development of knowledge (knowing), attitudes (knowing how to be) and skills (knowing how to do) that allow people to analyze the problems of their environment from an integrated vision [18], resulting in a relevant competence for any profession [19]. For Koerber and Osterhaus [20], the competence of complex thinking cannot be seen as a single skill or knowledge, since due to its breadth, it requires the acquisition of different subcompetencies that provide that broad and integrated vision.

Thus, the competence of complex thinking integrates four subcompetencies [7]:

(a) Systemic thinking that allows identifying the elements and interconnections that make up a problem or an environmental situation [21].
(b) Critical thinking to analyze and evaluate reasoning considering its validity and its differentiation with existing knowledge and structures [22].
(c) Scientific thinking, which provides methodologies that allow the construction of objective reasoning when making decisions [23].
(d) Innovative thinking, which provides creative and novel elements to evaluate the environment and the problems faced by the individual [24].

Social entrepreneurship competency is also made up of various elements, involving knowledge, attitudes and skills [25]. In general, social entrepreneurship competency enables the individual to devise and develop innovative solutions of social value, considering the importance of creativity in solving environmental problems [26,27]. Although there is a wide literature that points out different elements that can be associated with social entrepreneurship, this article is based on the proposal of García-González et al. [28], who frame this competency based on 4 subcompetencies and 17 indicators that show the cross-cutting characteristics of this competency (see Table 1).

**Table 1:** Social entrepreneurship competency and sub-competencies and indicators.

Sub-competencies	Indicators
Self-control	Motivation; perseverance and resilience; and tolerance to uncertainty and ambiguity
Leadership	Strategic planning; communication and persuasion; Mobilizing people; and collaborative work.
Social awareness and value	Social involvement; empathy; identification of social/environmental issues; orientation towards sustainability; and ethical sense
Social innovation and financial sustainability	Creativity; socio-economic and financial literacy; appraisal of ideas, results, and impacts on the environment and people; learning and adaptability; and management of limited resources for social projects

Thus, it is possible to appreciate that both competencies can be of high formative value for professionals of any discipline due to the transversality and relevance that contemporary universities give them [29,30].

### 2.3 Methodology

#### 2.3.1 Population and implementation

For this study, a convenience sample of 164 students (73 men and 91 women) from a technological university in the western region of Mexico comprised five groups for an educational intervention to develop a process of ideation for a social entrepreneurship project (Table 2). The implementation of this experimental study and the ethical implications involving individuals in this study was regulated and approved by the interdisciplinary research group R4C, with the technical support of the Writing Lab of the Institute for the Future of Education at Tecnologico de Monterrey.

**Table 2:** Characteristics of the sample.

Men		Women		Total	
*n*	*%*	*n*	*%*	*n*	*%*
73	45	91	55	164	100

The study was conducted in August and September 2022, during a five-week course in which first-semester students from various disciplinary areas participated. The implementation format was face-to-face, with the support of a facilitator who accompanied these groups during the educational intervention and the measurement of results.

#### 2.3.2 Instruments

Two validated instruments were used to diagnose and evaluate the social entrepreneurship and complex thinking competencies:

(a) Social Entrepreneurship: The Social Entrepreneur Profile instrument had 24 self-applicable questions evaluated with a Likert scale [31]. It assessed four sub-competencies: self-control, leadership, social awareness and value, and social innovation and financial sustainability (Table 3).

**Table 3:** Entrepreneurial profile. Instrument items.

Sub-competencies	Indicator	Item	Question
Self-control	Motivation	1	When I am passionate about something, I do my best to achieve my goals.
	Perseverance and resilience	2	When I am passionate about my work, I do my best to finish it, even if I face adverse circumstances, lack of time, or distractions.
	Perseverance and resilience	3	Despite rejection or problems, I always seek to achieve my goals.
	Tolerance to uncertainty	4	I am tolerant of ambiguous situations or situations that generate uncertainty.
Leadership	Strategic planning	5	I have the ability to establish a clear goal and the steps to achieve it.
	Communication and Persuasion	6	I am able to convince others of my ideas and actions.
	Communication and Persuasion	7	I master different ways of communicating my ideas: in writing, video, or face-to-face talks.
	Mobilizing People	8	I can delegate activities to my team members according to their profiles.
	Mobilizing People	9	I have the ability to identify the strengths and weaknesses of my co-workers.
	Collaborative Work	10	I am facilitated to collaborate actively in a team to achieve common goals.
Social Awareness and Social Value	Social Involvement	11	I am passionate about working for social causes.
	Social Involvement	12	I believe that my life’s mission is to work for social change and improve people’s lives.
	Empathy	13	I am interested in leading an initiative with favorable results for society and/or the environment.
	Identification of Social and Environmental problems.	14	I can identify problems in the social or environmental environment to generate innovative solutions.
	Identification of Social and Environmental problems	15	I am committed to participating in the social aspects of my environment.
	Orientation towards Sustainability	16	I believe that economic growth should occur with equal opportunities and equity for all.
	Ethical sense	17	My actions and behaviors are governed by moral standards based on respect and care for people and nature.
Social Innovation and Financial Sustainability	Creativity	18	I know how to apply strategies to create new ideas or projects.
	Socio-economic and financial literacy	19	I know how to apply accounting and financial knowledge to the development of an enterprise.
	Socio-economic and financial literacy	20	I have notions about logistics to carry out the management of an organization.
	Socio-economic and financial literacy	21	I know how to make a budget to achieve a project.
	Appraisal of ideas, results, and impacts on the environment and people.	22	I know how to establish evaluation criteria and measure social impact results.
	Learning and adaptability	23	I believe that making mistakes offers us new learning opportunities.
	Management of limited resources for social projects	24	I know strategies to develop a project, even with scarce resources.
(b) Complex thinking: The E-Complexity instrument aimed to measure the participants' perception of the level of mastery regarding the reasoning-for-complexity competency and its sub-competencies. The E-Complexity instrument has been theoretically and statistically validated by a team of experts [32]. The instrument consisted of 25 items divided into four sub-competencies: Scientific Thinking, Systemic Thinking, Innovative Thinking, and Critical Thinking (Table 4).

**Table 4:** E-Complexity. Instrument items.

Sub-competencies	Item	Question
Systemic Thinking	1	I can find associations between a project’s variables, conditions, and constraints.
	2	I identify data from my discipline and other areas contributing to solving problems.
	3	I participate in projects that need to be solved using inter/multidisciplinary perspectives.
	4	I organize information to solve problems.
	5	I enjoy learning different perspectives on a problem.
	6	I am inclined to use strategies to understand the parts and whole of a problem.
Scientific Thinking	7	I have the ability to identify the essential components of a problem to formulate a research question.
	8	I know the structure and formats for research reports used in my area or discipline.
	9	I identify the structure of a research article used in my area or discipline.
	10	I apply the appropriate analysis methodology to solve a research problem.
	11	I design research instruments consistent with the research method used.
	12	I formulate and test research hypotheses.
	13	I am inclined to use scientific data to analyze research problems.
Critical Thinking	14	I can critically analyze problems from different perspectives.
	15	I identify the rationale for my own and others' judgments to recognize false arguments.
	16	I self-evaluate the level of progress and achievement of my goals to make the necessary adjustments.
	17	I use reasoning based on scientific knowledge to make judgments about a problem.
	18	I make sure to review the ethical guidelines of the projects in which I participate.
	19	I appreciate criticism in the development of projects to improve them.
Innovative Thinking	20	I know the criteria to determine a problem.
	21	I have the ability to identify variables from various disciplines that can help answer questions.
	22	I apply innovative solutions to diverse problems.
	23	I solve problems by interpreting data from different disciplines.
	24	I analyze research problems considering the context to create solutions.
	25	I tend to evaluate the solutions to a problem with a critical and innovative sense.

#### 2.3.3 Compositional data analysis

Compositional data analysis (CoDa) focuses on studying data whose importance lies in the relative information between its parts and not in absolute values [33,34]. Generally, but not always, these data are expressed in a closed form, summing to a constant. Similarly, these data tend to be expressed as vectors of percentages, proportions, concentrations, and frequencies as appropriate [35]. Compositional data are strictly positive and range between 0 and the value of the constant (in case of closing to a constant). The above implies that the data are not free to oscillate independently. If one increases, the other decreases (constant sum constraint). This constraint forces a correlation coefficient to be negative in each row of the correlation matrix [8,36]. For this reason, the existence of a bias towards negative correlation could lead to spurious correlations. Eq. (1) identifies a compositional vector that has been closed to a constant K:

(1) $$X=C\left[x_{1},x_{2},x_{3},.\;.\;.,x_{D}\right]\text{,}$$

$$X=\left[\frac{x_{1}\;*\;k}{\sum_{i=1}^{D}x_{i}},\frac{x_{2}\;*\;k}{\sum_{i=1}^{D}x_{i}},\frac{x_{3}\;*\;k}{\sum_{i=1}^{D}x_{i}},.\;.\;.,\frac{x_{D}\;*\;k}{\sum_{i=1}^{D}x_{i}}\right]\;\text{.}$$

The sample space to which the compositional data belong is called *Simplex* (see Eq. (2)). The *Simplex* of D number of parts is a subset of the real space of dimension D. D of two parts is commonly represented as a line segment; D of three parts is represented as a triangle, and D of four parts is represented as a tetrahedron. Although the graphical representation is only possible for D equal to and less than four parts, it is possible to perform operations with a larger number of parts [35].

(2) $$S^{D}=\left\{x={\left(x_{1},.\;.\;.,x_{D}\right)}^{\prime }\varepsilon \;R^{D}\;|\;x_{i}\;>0,\sum_{i=1}^{D}x_{i}=K\right\}\text{.}$$

Ignoring the compositional nature of our data and using them in raw form with standard statistical tools could cause out-of-sample-space predictions, sub-compositional inconsistency, and spurious correlations [10,[37], [38], [39], [40], [41], [42]]. Other implications can be found in Aitchison [43,44], Chayes [45], and Pearson [46], among others.

To address the limitations of dealing with data of a compositional nature, Aitchison [44] proposed an algebraic-geometric structure for the Simplex (known as Aitchison geometry). In Aitchison geometry, data can be expressed in the form of coordinates formed by logarithms of the compositional parts (also known as the log-ratio approach). The use of coordinates based on log-ratios preserves the principles of data of compositional nature (e.g., such as scale invariance, permutation invariance and sub-compositional coherence).

In this sense, Aitchison [47,48] would develop the *additive-log-ratio (alr)* and the *centered-log-ratio (clr)*. Later, Egozcue et al. [49], introduced the *isometric-log-ratio (ilr)*, also known as *orthonormal-log-ratio (olr)*. The use of each of these coordinates will depend on the desired analysis, since each of them has distinctive features. Table 5 shows the *alr*, *clr*, and *ilr/olr* coordinates for a three-dimensional composite. Once the data are expressed as coordinates, the standard statistical tools can be used without problem.

**Table 5:** *Alr*, *clr,* and *ilr/olr* coordinates. Composition of three dimensions.

Composition	Coordinates		
	*alr*	*clr*	*ilr/olr*
X = [x_1_,x_2_,x_3_]	*alr(X)* = $\left[\ln\frac{x_{1}}{x_{3}},\ln\frac{x_{2}}{x_{3}}\right]$*.*	$clr\left(X\right)=$ [ $\ln\frac{x_{1}}{{\left(x_{1}*\;x_{2}*\;x_{3}\right)}^{1/3}}$ , $\ln\frac{x_{2}}{{\left(x_{1}*\;x_{2}*\;x_{3}\right)}^{1/3}}$ , $\ln\frac{x_{3}}{{\left(x_{1}*\;x_{2}*\;x_{3}\right)}^{1/3}}$].	$ilr\left(X\right)=[\;\frac{1}{\sqrt{2}}\ln\frac{x_{1}}{x_{2}}\text{,}$ $\frac{1}{\sqrt{6}}\ln\frac{x_{1}*x_{2}}{x_{3}^{2}}$].

Being a method based on logarithms, the presence of zeros and the absence of values in the composition is a problem. Depending on the nature of zeros and missing values, these can be imputed through different methods [[50], [51], [52]].

To carry out the compositional processing of the data, the following has been carried out. Firstly, each of the competencies has been divided into its respective sub-competencies. Secondly, each of the items measured by each of the sub-competencies was identified. From these items, the maximum total that can be obtained by each of the students per sub-competency has been obtained. In this sense, all students will have the same total score, but the distribution of points among the different sub-competencies will be different for each individual. In other words, our observations will have the same “size” but a different “shape”. This means that the relevance and importance of the study lies in the differences in the relative values (their “shapes”). In this sense, Greenacre [53] states that one way to focus on the “shape” of observations while avoiding problems of scale caused by the “size” of our observations is through the log-ratio approach (i.e., compositional data analysis) for strictly positive data. In this context, each of the items comprising the sub-competencies of complex thinking and social entrepreneurship have been added and each of the sub-competencies has been assigned a weight of 0.25 (see Eq. (3)). Subsequently, the total of each observation (compositional vector) has been scaled so that the sum of its four sub-competencies adds up to 100%.

(3) $$C=\left[\;\left({Sc}_{1}*w_{1}\right),.\;.\;.,\left({Sc}_{D}*w_{D}\right)\;\right],\sum_{i=1}^{D}{Sc}_{i}=C.$$

In this sense, this study’s data set of complex thinking and social entrepreneurship competencies correspond to compositional data samples. Each compositional vector represents a student, and the different compositional parts correspond to the sub-competencies of each competency (see Table 6, Table 7).

**Table 6:** Complex thinking. Compositional parts.

Complex Thinking			
1 Scientific thinking	2 Critical thinking	3 Innovative thinking	4 Systemic thinking

**Table 7:** Social entrepreneurship. Compositional Parts.

Social Entrepreneurship			
1Self-control	2Social awareness and social value	3Social innovation and financial Sustainability	4Leadership

The analysis of high-dimensional data can give place to multicollinearity problems. To avoid the latter, principal component analysis (PCA) creates a new set of uncorrelated variables that capture the maximum explained variability of the original data. It is worth mentioning that there will be as many principal components as the number of variables being analyzed [54]. The PCA was performed through *clr* coordinates (see Eq. (4)). The *clr* coordinates are obtained by dividing each component by its geometric mean and then applying the logarithm [36]. In Eq. (4) the geometric mean is considered per row (i.e., per sample). Among the benefits of these coordinates is their use in exploratory analysis, such as PCA and *clr*-biplots [55].

(4) $$clr\left(X\right)=\left[\ln\frac{x_{1}}{g\left(x\right)},\ln\;\frac{x_{2}}{g\left(x\right)},.\;.\;.,\ln\;\frac{x_{D}}{g\left(x\right)}\right]\text{,}$$

$$X=\left(x_{1},.\;.\;.,x_{D}\right)\text{,}$$

$$g\left(X\right)={\left(\prod_{i=1}^{D}x_{i}\right)}^{1/D}\;\text{.}$$

#### 2.3.4 Cluster analysis

Cluster analysis groups similar observations according to specific criteria [56]. Thus, observations in the same cluster are similar, and they differ from those in other groups. For this study, Ward’s hierarchical clustering method (i.e., a bottom-up agglomerative method) based on the sum of squares criterion was applied [57].

Balanced *ilr/olr* coordinates were used to carry out this analysis. Balances represent a particular form of orthonormal-based *ilr/olr* coordinates that help to better interpret the coordinates by explaining the relative variation between groups of composition parts [58]. It is worth mentioning that the Euclidean distances calculated from *ilr/olr* coordinates are equivalent to the distances defined in Aitchison [48]. Sequential Binary Partition (SBP) was used to obtain these balances. Table 8, Table 9 show the partition of the competencies into four parts. This method aims to divide the set of parts of a composition into two groups of parts. The groups generated must be divided again into two groups of parts, and so on, until we obtain groups of only one part. The resulting number of parts will be D-1. The parts of the groups are coded as +1 and −1 (+1 corresponds to the numerator and −1 to the denominator.). The value zero means that the part has not been taken for partitioning. Descriptively, Eq. (5) shows how to obtain the *ilr/olr* coordinates. The *ilr/olr* coordinates resulting from the partition and used in this study are shown in Eqs. (6a), (6b).

**Table 8:** Complex thinking. Sequential binary partition.

Complex Thinking				
Order	X_1_ (Scientific T.)	X_2_ (Critical T.)	X_3_ (Innovative T.)	X_4_ (Systemic T.)
1	+1	+1	−1	−1
2	−1	+1	0	0
3	0	0	−1	+1

**Table 9:** Social entrepreneurship. Sequential binary partition.(5)$x_{i}^{*}=\ln\;\frac{{\left(\Pi_{+}\;x_{j}\right)}^{a_{+}}}{{\left(\Pi_{-}\;x_{k}\right)}^{a_{-}}}\text{,}$

Social Entrepreneurship				
Order	X_1_ (Self-control)	X_2_ (Social awareness and social value)	X_3_ (Social Innovation and Financial Sustainability)	X_4_ (Leadership)
1	−1	+1	+1	−1
2	0	−1	+1	0
3	+1	0	0	−1

$a_{+}=\sqrt{\frac{s}{r\left(r+s\right)}}a_{-}=\sqrt{\frac{r}{s\left(r+s\right)}}a_{0}=0$.

Where:

$\Pi_{+}$ y $\Pi_{-}$. Corresponds to the products of the parts coded as +1 and −1, respectively, in partition i.
r = number of positive codes.
S = number of negative codes.

(6a) $$X_{1CT}^{*}=\ln\;\frac{{\left(Scientific\;T.\cdot Critical\;T.\right)}^{\sqrt{1/4}}}{{\left(Innovative\;T.\cdot Systemic\;T.\right)}^{\sqrt{1/4}}},X_{2CT}^{*}=\ln\;\frac{{\left(Critical\;T.\right)}^{\sqrt{1/2}}}{{\left(Scientific\;T.\right)}^{\sqrt{1/2}}}X_{3CT}^{*}=\ln\;\frac{{\left(Systemic\;T.\right)}^{\sqrt{1/2}}}{{\left(Innovative\;T.\right)}^{\sqrt{1/2}}}$$

(6b) $$X_{1SE}^{*}=\ln\;\frac{{\left(\;Awar.\;and\;S.V.\cdot Innov.\;and\;F.\;sust.\right)}^{\sqrt{1/4}}}{{\left(Self\;control\cdot Leadership\;\right)}^{\sqrt{1/4}}},X_{2SE}^{*}=\ln\;\frac{{\left(Innov.\;and\;F.\;sust.\right)}^{\sqrt{1/2}}}{{\left(\;Awar.\;and\;S.V.\right)}^{\sqrt{1/2}}},X_{3SE}^{*}=\ln\;\frac{{\left(Self\;controll\right)}^{\sqrt{1/2}}}{{\left(Leadership\;\right)}^{\sqrt{1/2}}}\text{.}$$

The interpretation of the *ilr/olr* coordinates can be straightforward or complicated depending on the number of parts and the expertise of the researcher. For some authors the interpretation of the coordinates is one of the main drawbacks of this method. For ease the interpretation, the $X_{1CT}^{*}$ coordinate is explained as follows. This coordinate indicates that scientific and critical thinking is given more value than systemic and innovative thinking. That is, if this coordinate is positive for a certain student, it would indicate that the student has the ability to evaluate and solve problems beyond existing paradigms, in addition to the fact that the solution would be based on objective and validated methodologies. This is above creative solutions or solutions based on seeing the problem from different interconnected factors. On the other hand, coordinate $X_{2CT}^{*}$ indicates that more importance is being given to critical thinking than to scientific thinking. In this sense, if this coordinate has a positive value in any of the students, it might indicate that this person seeks to evaluate reality and existing information, discerning what is not said or what can be put in a different way. This is over and above analysis based on objective and standardized methods (i.e., scientific thinking). As far as coordinate $X_{3CT}^{*}$ is concerned, it would indicate that in the case of being positive, the student would be developing a greater degree of systemic thinking than innovative thinking. This means that the student would be developing to a greater extent the capacity to analyze problems and provide solutions in an integrated way, from an inter- and transdisciplinary perspective. This is in comparison to mental processes of seeking original solutions (i.e., innovative thinking).

On the other hand, the *ilr/olr* coordinates of social entrepreneurship would indicate the following. The $X_{1SE}^{*}$ coordinate indicates that more importance is given to social awareness and social value, and to innovation and financial sustainability over self-control and leadership. In other words, priority is given to working for social causes with administrative and management skills over passion and leadership. The $X_{2SE}^{*}$ coordinate suggests that innovation and financial sustainability are given more importance over social awareness and social value. In other words, more importance is given to strategies for creating new projects and entrepreneurship over the creation of initiatives with social impact. Finally, the co-ordinate $X_{3SE}^{*}$ explains that self-control is more important than leadership. In other words, the passion and ambition of the students to achieve their goals is of greater interest than the fact of delegating or convincing others to carry out actions.

#### 2.3.5 Regression analysis

Regression analysis was used to find significant associations between sub-competencies of complex thinking and social entrepreneurship competencies. The *ilr/olr* coordinates are real variables and allow the use of standard linear regression (see Eq. (7)). For the analysis, complex thinking sub-competencies have been considered as covariates (Eq. (6a)), and, as response variables, social entrepreneurship sub-competencies were considered (Eq. (6b)). To indicate significance, a p-value less than or equal to 0.05 has been taken into account.

(7) $$x_{i}^{*}=b_{0}+\sum_{k=1}^{r}\left(m_{ik}\cdot B_{k}\right)+e_{i},i=1,.\;.\;.,n\text{.}$$

The statistical analyses were performed through the use of R [59], Rstudio [60], and CodaPack [61]. CodaPack is a software tool focused on the processing of compositional data.

**Fig. 1:**
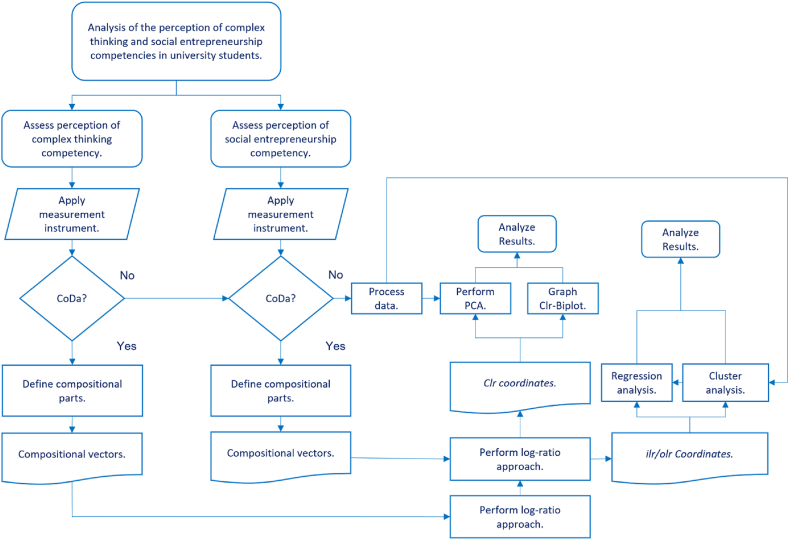
Methodological flow diagram. Source: Created by the authors.

Fig. 1 shows the methodological diagram which illustrates the steps followed to carry out this research.

## 3 Results and discussion

**Fig. 2:**
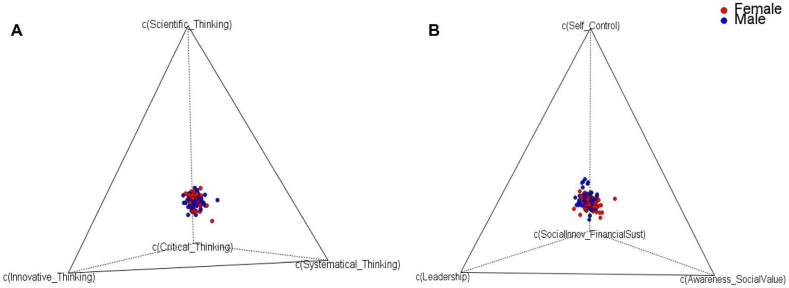
Sample compositional vectors in the *Simplex*: A) Complex Thinking, B) Social Entrepreneurship. Source: Created by the authors through CodaPack [61].

Fig. 2 shows the *Simplex* of the compositional vectors of each competency analyzed as a first exploratory analysis. In the *Simplex*, the sample space to which the compositional vectors of each competency analyzed (i.e., Complex Thinking and Social Entrepreneurship) belong is illustrated. Each point in the *Simplex* represents a student, and each student has been color-coded by gender. Moreover, each vertex of the tetrahedron (four-part *Simplex*) corresponds to the sub-competencies for the perception of Complex Thinking and Social Entrepreneurship (see Table 6, Table 7, respectively).

It is clear from Fig. 2(A and B) that the data analyzed correspond to compositional data, that the sample space to which they belong is the *Simplex*, and that the importance of these data lies in the relative information (i.e., information between their parts). Likewise, interpreting the data relationships in the *Simplex* sample space is complicated. For the correct analysis, it is necessary to analyze the data using an appropriate geometric structure with appropriate operations and distances to the sample space. In this regard, it is essential to express the data in *clr* or *ilr/olr* coordinates (based on the type of analysis), which function as real vectors with which data analysis can be performed using Euclidean geometry and to which standard statistical tools can be applied.

The PCA is performed on the data transformed into *clr* coordinates for each variable (competencies) with their respective sub-competencies. Regarding the complex thinking competency (Table 10), we observe that the total variance explained by the first principal component (PC1) and the second principal component (PC2) is 43% and 32%, respectively, a cumulative proportion of 75%. It is observed that PC1 has a positive correlation with the sub-competency of scientific thinking. This could imply that this component explains students' perception concerning their capacity for decision making and their ability to solve problems using, for example, hypotheses and objective and validated methodologies. On the other hand, PC2 is positively correlated with the sub-competency of innovative thinking. This could imply that PC2 explains the students' perception of their ability to evaluate reality from different angles and seeking to generate different and feasible ideas and proposals.

**Table 10:** Principal component analysis matrix: Complex Thinking.

	PC1	PC2	PC3
Clr-Scientific thinking.	0.67	−0.50	0.19
Clr-Critical thinking.	−0.56	−0.00	0.65
Clr-Innovative thinking.	0.27	0.81	−0.13
Clr-Systemic thinking.	−0.38	−0.30	−0.71
Cum.Prop.Expl.	0.43	0.75	1.00

Regarding the students' perception of social entrepreneurship competency, Table 11 shows the principal component analysis performed. Table 11 shows that the total variance explained by PC1 and PC2 is 80% (50% and 30%, respectively). Likewise, it can be observed that PC1 is correlated with the sub-competency of social innovation and financial sustainability. In this sense, this component would explain students' perception of the accounting and financial knowledge capacity for developing an enterprise with social impact. On the other hand, PC2 is mainly correlated with the sub-competency of social awareness and social value. In this sense, PC2 would explain the students' perception of their sense of ethics, social implications, identification of social problems, and commitment to solving social and environmental problems.

**Table 11:** Principal component analysis matrix. Social Entrepreneurship.

	PC1	PC2	PC3
Clr-Self-control.	0.19	−0.55	0.64
Clr-Social awareness and social value.	0.41	0.74	0.12
Clr-Social Innovation and financial sustainability.	−0.85	0.13	−0.01
Clr-Leadership.	0.24	−0.33	−0.75
Cum.Prop.Expl.	0.50	0.80	1.00

**Fig. 3:**
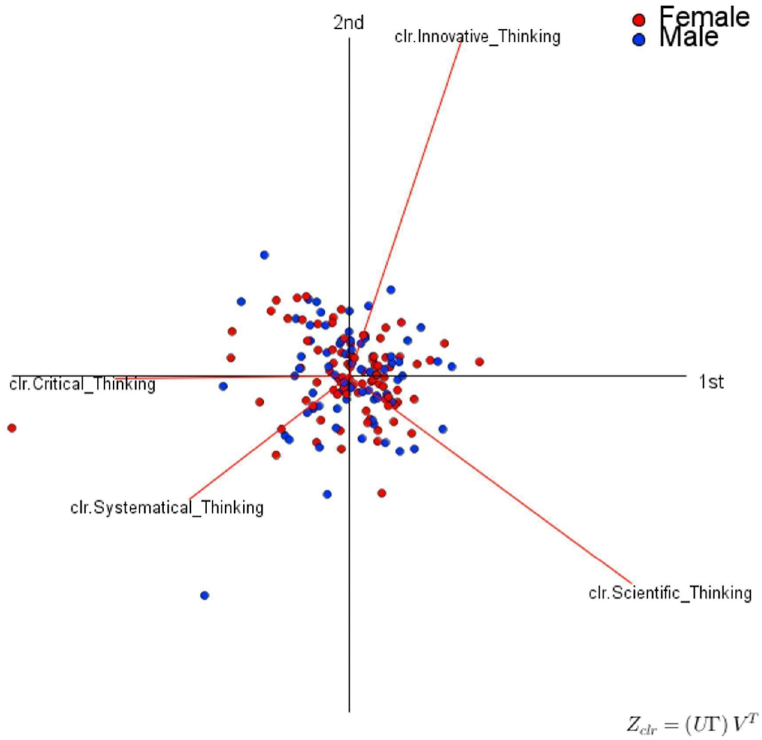
Complex Thinking. Clr-Biplots (α = 1). Source: Created by the authors.

**Fig. 4:**
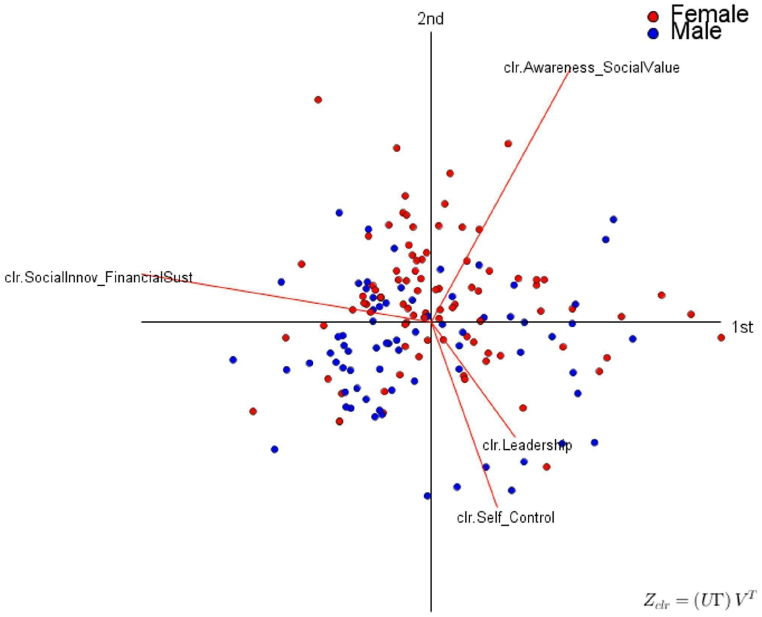
Social Entrepreneurship. Clr-Biplots (α = 1). Source: Created by the authors.

The capture by the first two principal components of most of the variability of the data allows a projection from the first two components without losing too much information. In this sense, the PCA results are represented in *clr*-Biplots (Fig. 3, Fig. 4), which favors the visualization of the observations (i.e., α = 1) for each competency.

Fig. 3 shows the form clr-Biplot (α = 1) regarding the perception of complex thinking in students color-coded by gender. It shows that the perception of both males and females concerning complex thinking is very similar except for two students who stand out specifically in the perception of *clr*-Critical Thinking and *clr*-Systemic thinking. Likewise, there are students whose perception of the sub-competencies of *clr*-Innovative thinking and *clr*-Critical thinking is good, and at the same time, they perceive themselves negatively in *clr*-Scientific thinking.

Fig. 4 shows the clr-Biplot of form (α = 1) regarding the students' perception of the social entrepreneurship competency, color-coded by gender. Fig. 4 shows a very particular behavior of male students concerning their perception of the sub-competencies *clr*-Social Innovation and Financial Sustainability, the sub-competency *clr*-Leadership, and the sub-competency *clr*-Self-Control. In these three sub-competencies, the perception of male students is very high. In this sense, when they are passionate about something, they tend to set a clear goal, lead, and achieve it through various adverse circumstances. It also indicates that male students perceive themselves as having the financial and accounting knowledge to develop an enterprise even when resources are scarce. On the other hand, Fig. 4 highlights the high perception of women for the sub-competency of *clr*-Social awareness and value. This means that they perceive themselves as having a high capacity to work on social causes, improve people's lives, and work for society and the environment. Likewise, women perceive themselves as low in the sub-competencies of *clr*-Leadership and *clr*-Self-control.

According to Gupta et al. [62], there is a very marked gender gap within traditional entrepreneurship, which makes males more representative than their female peers. However, this difference is blurred when discussing social entrepreneurship, in which women have balanced participation with male entrepreneurs. Even so, and according to Dickel and Eckardt [63], a large part of the social entrepreneurship proposals put forward by women never become a reality due to the lack of financing opportunities usually experienced by women for their ventures. Ultimately, women are underrepresented as participants or collaborators in entrepreneurship processes, which may influence their perception of leadership in contrast to their male peers [64].

A cluster analysis was performed to find patterns in the students' perception of complex thinking and social entrepreneurship competencies. As explained above, the *ilr/olr* coordinates (Equation 6a-b) have been used for the hierarchical cluster analysis considering Ward's sum of squares as a criterion. It should be recalled that the transformation of our data into coordinates is done in cluster analysis to make distances meaningful. In their natural form, these data cannot be used in statistical tools and need to be transformed to a real scale using the *ilr/olr* transformation.

Based on the dendrogram generated from the cluster analysis, it was decided to perform the cut-off and classify the students into four groups (clusters) for each of the variables studied (competencies). Therefore, each student is associated with a different group of the four groups generated for each competency.

Table 12 shows the number of students associated with each cluster concerning their perception of the competency of complex thinking. It is observed that most of the students were classified in cluster two (about 85 students), followed by cluster three (48 students), while groups one and four had the least number of students (29 and 2 students, respectively).

**Table 12:** Complex thinking. Cluster analysis.

Cluster	Women		Men		Total	
#	n	%	n	%	n	%
1	14	48	15	52	29	100
2	50	59	35	41	85	100
3	26	54	22	46	48	100
4	1	50	1	50	2	100

**Fig. 5:**
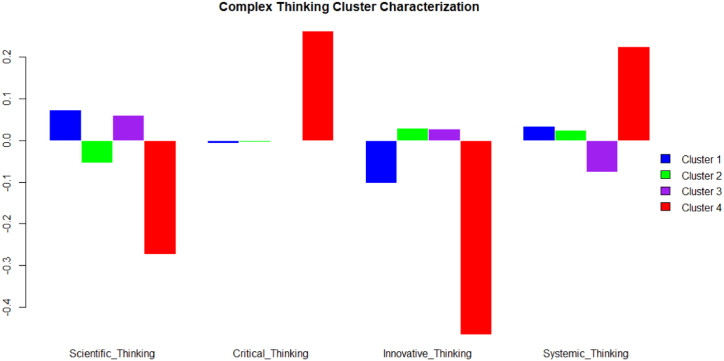
Complex thinking. Characterization of clusters. Geometric-mean bar plot. *Source:* Created by the authors.

Fig. 5 shows the characterization of the clusters concerning the students' perception of the development of the complex thinking sub-competencies through a geometric-mean bar plot [65]. It is observed that cluster one stands out for students who are perceived to be high in the development of the sub-competencies of scientific thinking and systematic thinking. Likewise, the students in this cluster are characterized by perceiving themselves as having a low level of development in the sub-competencies of critical and innovative thinking. On the other hand, students in cluster two are perceived to have a positive development of the sub-competencies of systemic and innovative thinking, while they are perceived to have a lower development in the sub-competencies of scientific and critical thinking. In cluster three, on the other hand, students perceive themselves as having a positive development in the sub-competencies of scientific and innovative thinking, while they perceive themselves as having a lower development in the sub-competencies of critical and systemic thinking. Finally, students in cluster four stand out for perceiving themselves as highly developed in the sub-competencies of critical and systemic thinking, and with a lower perception of achievement in the sub-competencies of scientific and innovative thinking.

On the other hand, Table 13 shows the number of students associated with each cluster concerning their perception of social entrepreneurship competency. It is observed that most students have been classified in clusters one and three (84 and 41 students, respectively), while clusters two and four have the lowest number of students (32 and 7, respectively).

**Table 13:** Social entrepreneurship. Cluster analysis.

Cluster	Women		Men		Total	
#	N	%	n	%	n	%
1	55	65	29	35	84	100
2	10	31	22	69	32	100
3	25	61	16	39	41	100
4	1	14	6	86	7	100

**Fig. 6:**
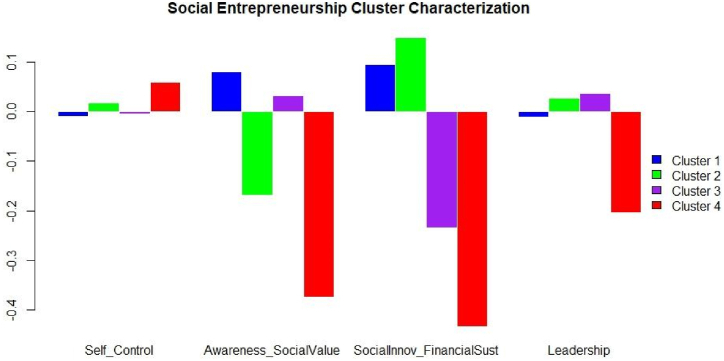
Social entrepreneurship. Cluster characterization. Geometric-mean bar plot. Source: Created by the authors.

Fig. 6 shows the characterization of the clusters formed concerning students' perception of social entrepreneurship sub-competencies through a geometric-mean bar plot [65]. Fig. 6 shows that students in cluster one perceived themselves to be high in two of the four sub-competencies of social entrepreneurship competency, i.e., awareness and social value, and social innovation and financial sustainability. On the other hand, students in cluster two perceived themselves highly in three of the four sub-competencies (self-control, social innovation and financial sustainability, and leadership). Students in cluster two perceive themselves as particularly deficient in the development of the sub-competency of awareness and social value. As for cluster three, this is characterized by students who perceive themselves to be high in the development of the sub-competencies of leadership and, awareness and social value. Likewise, the students in this cluster perceive themselves as having a low level of development of the sub-competencies of self-control and, social innovation and financial sustainability. On the other hand, within cluster four, students stand out for perceiving themselves as low in the achievement of the sub-competencies of leadership, social innovation and financial sustainability, and social awareness and value. Likewise, in this cluster there are also students who perceive themselves as high in the achievement of the sub-competency of self-control.

It is worth noting that Table 13 highlights cluster one, made up mainly of 65% women. This cluster is mainly made up of women with a high perception in the development of the sub-competencies of awareness and social value and social innovation and financial sustainability, and a low level of development with respect to the leadership sub-competency. (see Fig. 6). In this sense, Arredondo [64] and, Dickel and Eckard [63], point out that women tend to have a positive desirability, intentions in the development of enterprises with a very strong social component. On the other hand, these authors point out that women tend to feel undervalued and underrepresented in the process of entrepreneurship, which affects their leadership. In the latter, the results are consistent with those shown their studies.

Likewise, the behavior of cluster four, mainly composed of men (89%), is striking (see Table 13). The men in this cluster are perceived as having a low level of development in all the sub-competencies of social entrepreneurship except for the sub-competency of self-control (see Fig. 6). This means that men perceive themselves to be highly motivated and perseverant to undertake, but their projects may not prosper because they lack the rest of the sub-competencies.

Finally, an analysis was carried out to find any relationship between the perception of social entrepreneurship competency and complex thinking, especially its sub-competencies. To achieve the above, we performed a regression analysis. The ilr/olr coordinates corresponding to the sub-competencies of social entrepreneurship (Eq. (6b)) were considered as explanatory variables (covariates). In contrast, the *ilr/olr* coordinates corresponding to the sub-competencies of complex thinking (Eq. (6a)) were considered as response variables.

It is worth mentioning that to be congruent with the method and the nature of the data, we considered the compositional nature of the data in the response variables and covariates. Likewise, it is essential to point out that the model's predictive capacity is not the focus of these results but rather the significance of the covariates influencing the analyses was used in order to depict the association between the complex thinking sub-competencies and social entrepreneurship sub-competencies.

The analysis found a significant association between the *ilr/olr* coordinates of complex thinking sub-competencies and the *ilr/olr* coordinates of social entrepreneurship sub-competencies (Table 14). As regards the balance coordinate x*_1CT_ (first model at the top of Table 14), there is a significant association between the scientific and the critical thinking sub-competencies (in the numerator of the x*_1CT_ coordinate) with the social awareness and social value, self-control, and leadership sub-competencies (in the denominators of the x*_1SE_ and x*_2SE_ coordinates). The correspondence between numerators and denominators stems from the negative sign of the statistically significant beta coefficients.

**Table 14:** Regression analysis of the perception of complex thinking and social entrepreneurship competencies.

Model x*_1CT_	Β	t	*P*	VIF
x*_1SE_	−0.097	−2.000	0.047	1.047
x*_2SE_	−0.121	−2.937	0.003	1.496
x*_3SE_	0.003	0.060	0.950 (ns)	1.005
Model x*_2CT_	Β	t	*P*	VIF
x*_1SE_	−0.120	−2.051	0.041	1.047
x*_2SE_	−0.127	−2.544	0.011	1.049
x*_3SE_	−0.215	−2.892	0.004	1.005
Model x*_3CT_	Β	t	*P*	VIF
x*_1SE_	−0.127	−2.355	0.019	1.047
x*_2SE_	−0.114	−2.460	0.015	1.049
x*_3SE_	0.050	0.727	0.468 (ns)	1.005
Model Fit	R	R^2^	F	*p*
Model x*_1CT_	0.089	0.072	5.26	0.001
Model x*_2CT_	0.122	0.106	7.44	0.000
Model x*_3CT_	0.086	0.069	5.043	0.002

Regarding the balance coordinate x*_2CT_ (second model shown in Table 14), there is a significant association between critical thinking sub-competency (in the numerator of the x*_2CT_ coordinate) with the self-control, leadership, and social awareness and social value sub-competencies (in the denominators of the x*_1SE_, x*_2SE_ and x*_3SE_ coordinates).

Finally, the balance coordinate x*_3CT_ (third model shown in Table 14) show a significant association between systemic thinking sub-competency (in the numerator of the x*_3CT_ coordinate) with the social awareness and social value, self-control, and leadership sub-competencies (in the denominators of the x*_1SE_ and x*_2SE_ coordinates).

## 4 Conclusions

Although the primary finding of this article is the identification of a statistically significant association between the relative importance of complex thinking and social entrepreneurship sub-competencies, additional findings can be noted that show not only the relevance of its results but also the use of the compositional data methodology in the educational field. The possibility of being able to analyze the parts, components, and variables beyond the general results has made it possible to identify tendencies on the part of the population towards certain sub-competencies, responding to variables of the environment and the cultural imaginary due to the influence of the gender of the participating students.

It is recognized that one of the important limitations of this study is the impossibility to draw conclusions about the overall levels of the competencies. This means that only the relative importance of sub-competencies is studied. Moreover, the small sample size could somewhat limit the present study. In addition to the foregoing, it is acknowledged that it would have been interesting to carry out the analysis by type of discipline of the students. However, students from all disciplines were not available. Nevertheless, the data obtained, and the use of the methodology provide academically valuable information. In particular, we believe that highlighting the gender variable is particularly interesting given the context in which the study was carried out (i.e., in a Mexican university).

On a practical level, this article contributes in two ways. First, it allows us to see results that argue the relevance for educational institutions to work on the acquisition and development of competencies more efficiently and jointly. Second, this article introduces the methodology of compositional data analysis for competency measurement to the academic literature, expanding the possibilities of analysis and considering more elements that can influence educational processes.

Thus, the originality of this article is not only focused on the results that show a statistically significant relationship between social entrepreneurship and complex thinking competencies but also on the use of a methodology that has not been considered for the study of competencies in educational contexts, opening vast possibilities to rethink previously conducted analyses that can be enriched with the adoption of this method.

## Author contribution statement

Marco Cruz-Sandoval, Ph.D.: Conceived and designed the experiments; Performed the experiments; Analyzed and interpreted the data; Contributed reagents, materials, analysis tools or data; Wrote the paper.

José Carlos Vázquez-Parra; Martina Carlos-Arroyo: Conceived and designed the experiments; Performed the experiments; Contributed reagents, materials, analysis tools or data; Wrote the paper.

## Funding statement

This manuscript is a product of the financial support from the Tecnologico de Monterrey through the funds: EduToolkit: Innovation with artificial intelligence for the development of social entrepreneurship, innovation and complex thinking skills [NOVUS ID 206 and NOVUS ID 268], and the Challenge-Based Research Funding Program 2022 [Project ID # I003 - IFE001 - C2-T3 - T].

## Data availability statement

Data will be made available on request.

## Declaration of interest’s statement

The authors declare no conflict of interest.
